# Suicide of Dr. Wells

**Published:** 1848-02

**Authors:** 


*Suicide of Dr. Wells.*—The papers announce the death of Dr. Wells of Hartford, Conn., under melancholy circumstances. It appears that Dr. W. from frequent inhalations of the Chloroform, for its exhilirating effect, induced a species of insanity leading him into eccentricities of conduct for which he was arrested and committed to the *tombs* in the city of New-York. The mortification arising from his imprisonment, it is supposed, impelled him to commit suicide. Dr. Wells is known as the discoverer of the application of anæsthetic inhalations to surgical operations.

